# At a glance: the largest Niemann-Pick type C1 cohort with 602 patients diagnosed over 15 years

**DOI:** 10.1038/s41431-023-01408-7

**Published:** 2023-07-11

**Authors:** Pilar Guatibonza Moreno, Luba M. Pardo, Catarina Pereira, Sabine Schroeder, Deepthi Vagiri, Ligia S. Almeida, Carlos Juaristi, Heba Hosny, Clarice C. Y. Loh, Anika Leubauer, Galina Torres Morales, Sebastian Oppermann, Marius-Ionuț Iurașcu, Steffen Fischer, Tara-Marisa Steinicke, Nikenza Viceconte, Claudia Cozma, Krishna Kumar Kandaswamy, Jorge Pinto Basto, Tobias Böttcher, Peter Bauer, Aida Bertoli-Avella

**Affiliations:** 1 CENTOGENE GmbH, Rostock, Germany; 2 Univesrity of Rostock, Rostock, Germany

**Keywords:** Metabolic disorders, Medical genomics

## Abstract

Niemann-Pick type C1 disease (NPC1 [OMIM 257220]) is a rare and severe autosomal recessive disorder, characterized by a multitude of neurovisceral clinical manifestations and a fatal outcome with no effective treatment to date. Aiming to gain insights into the genetic aspects of the disease, clinical, genetic, and biomarker PPCS data from 602 patients referred from 47 countries and diagnosed with NPC1 in our laboratory were analyzed. Patients’ clinical data were dissected using Human Phenotype Ontology (HPO) terms, and genotype–phenotype analysis was performed. The median age at diagnosis was 10.6 years (range 0–64.5 years), with 287 unique pathogenic/likely pathogenic (P/LP) variants identified, expanding *NPC1* allelic heterogeneity. Importantly, 73 P/LP variants were previously unpublished. The most frequent variants detected were: c.3019C > G, p.(P1007A), c.3104C > T, p.(A1035V), and c.2861C > T, p.(S954L). Loss of function (LoF) variants were significantly associated with earlier age at diagnosis, highly increased biomarker levels, and a visceral phenotype (abnormal abdomen and liver morphology). On the other hand, the variants p.(P1007A) and p.(S954L) were significantly associated with later age at diagnosis (*p* < 0.001) and mildly elevated biomarker levels (*p* ≤ 0.002), consistent with the juvenile/adult form of NPC1. In addition, p.(I1061T), p.(S954L), and p.(A1035V) were associated with abnormality of eye movements (vertical supranuclear gaze palsy, *p* ≤ 0.05). We describe the largest and most heterogenous cohort of NPC1 patients published to date. Our results suggest that besides its utility in variant classification, the biomarker PPCS might serve to indicate disease severity/progression. In addition, we establish new genotype–phenotype relationships for “frequent” *NPC1* variants.

## Introduction

Niemann-Pick type C1 disease (NPC1 [OMIM 257220]) is a rare genetic disorder presenting numerous neurovisceral clinical manifestations. Although the pathophysiology of NPC is complex, the most well-accepted hypothesis postulates that an intracellular disruption of the cholesterol transport causes accumulation of unesterified cholesterol and other molecules in the late endosomal/lysosomal compartments. Consequently, the abnormal accumulation of these substances leads to damage and degeneration of various cells and tissues of the body, in particular the neurons in the central nervous system [1, 2].

The clinical presentation of NPC1 is heterogeneous and usually includes neurodevelopmental delay, cognitive impairment, ataxia, abnormal ocular movements, psychosis, mood disorders, dementia, and hepatosplenomegaly. In addition, the age of onset of clinical symptoms is highly variable and can range between the neonatal period through adulthood. Furthermore, age of onset of neurological symptoms directly correlates to life expectancy and could be used to predict disease severity [3, 4]. The manifestations in the perinatal period and infancy (<2 years) are predominantly visceral symptoms such as hepatosplenomegaly and jaundice. From late infancy onwards, the clinical presentation is dominated by neurologic manifestations (2 to 6 years). Younger children may present with hypotonia and developmental delay, with subsequent emergence of ataxia, dysarthria, dysphagia, and in some children, epileptic seizures, dystonia, and gelastic cataplexy. Older children (>6 years) and adults may present predominantly with apparent early-onset dementia or psychiatric manifestations [5–7].

NPC1 is inherited as an autosomal recessive disease and is caused by the biallelic pathogenic or likely pathogenic (P/LP) variants in the *NPC1* gene [8, 9]. To date, 651 (likely) causative genetic variants in the *NPC1* gene are accountable for the disease [10]. A similar but ultra-rare disorder known as NPC2 [OMIM 607625] it is caused by P/LP variants in the *NPC2* gene [8, 11]. NPC2 and NPC1 proteins sequentially interact and mediate the egress of cholesterol from the endolysosomal system [2, 12].

Establishing the diagnosis of NPC and prediction of disease course is challenging given the heterogeneous clinical presentations, variable age of onset, and allelic heterogeneity of the disease [13]. Genetic testing includes targeted *NPC1/NPC2* analysis, or dedicated gene panels, which can be combined with enzymatic assay testing of acidic sphingomyelinase and the biomarker N-palmitoyl-O-phosphocholineserine (PPCS, formerly known as lyso-SM-509) [14, 15].

With this work, we describe the largest cohort of genetically diagnosed NPC1 patients reported to date. The analysis of our Biodatabank included clinical, genetic, and biomarker results from this group of 602 NPC1 patients. Our results expand the allelic heterogeneity of *NPC1*, including the report of 73 novel causative variants, and their geographical aggregation. Furthermore, we disclose novel genotype-HPO terms-biomarker relationships and illustrate the value of combined genetic/biomarker testing to diagnose and increase the understanding of NPC1.

## Methods

The current project has been conducted within a diagnostic setting, and in the second step, utilized deidentified data and samples—thus not requiring Institutional Review Boards (IRB) approval.

We queried the CENTOGENE Biodatabank for unrelated (consecutive) patients who received a genetic diagnosis of NPC1 based on the detection of biallelic P/LP *NPC1* variants between October 2006 and March 2021. Information was compiled related to age at diagnosis, gender, and country of origin along with available clinical, genetic, and biomarker data. The clinical information provided by referring clinicians was converted into HPO terms by a dedicated team of scientists. During this process, the affection status of the individual, age at onset, and family history, as well as the curated HPO terms were registered in our laboratory management system (LIMS) and the CENTOGENE Biodatabank. Every provided document was carefully inspected, and clinical terms were marked and then stored for quality control purposes. In cases of contradictory or unclear information, the referring clinician was contacted for clarification.

DNA was extracted using standard methods, usually from dried blood spots (DBS) submitted on filter cards (CentoCard^®^) (Table 1). Sanger sequencing was done on a 3730xl sequencer (Thermo Fisher Scientific, Waltham, MA). Primers to cover the 25 exons and intron/exon boundaries of *NPC1* (NM_000271.4) were available on request. MLPA^®^ analyses were performed with commercially available kits according to manufacturer’s instructions (MRC-Holland, Amsterdam, The Netherlands, Probemix P193-B3). MLPA reactions were run in ABI 3730xl/3130xl DNA Analyzers (Applied Biosystems).

**Table 1:** Characteristics of the cohort of 602 NPC1 patients.

Features	Cohort of 602 patients	
Gender	*N*	%
Male	298	49.5
Female	257	42.7
Unknown	47	7.8
Age at diagnosis	Range 0–65 years	
0–5 years old	266	44.2
>5–18 years old	136	22.6
>18 years old	91	15.2
Unknown	109	18
Geographical origin (region)		
North America	2	0.3
Latin America	118	19.6
Europe	67	11.1
Middle East	190	31.6
Africa	89	14.8
Asia	63	10.5
Unknown	73	12.1
Clinical information		
Yes (HPOs)	456	75.7
No CI provided	146	24.3
Type of sample		
Dried blood spots	466	77.4
Blood	52	8.6
DNA	19	3.2
Skin	1	0.2
Amniotic fluid, CV	6	1.0
Buccal swab	1	0.2
Multiple type of sample	57	9.5
Genetic testing		
Targeted gene (Sanger)	244	40.5
Targeted gene (NGS)	62	10.3
NPC1 MLPA/qPCR/Sanger	7	1.2
Panel	214	35.5
Exome sequencing	67	11.1
Genome sequencing	8	1.3
Total	602	100.0

### Panel, exome sequencing (ES), and genome sequencing (GS)

For the CentoMetabolic® panel, the coding regions, 10 bp of flanking intronic sequences, and known P/LP variants (coding and non-coding) of the selected genes, including *NPC1*, were targeted for analysis [16]. Data analysis, including alignment to the hg19 human reference genome (Genome Reference Consortium GRCh37), variant calling, and annotation was performed using validated in-house software. ES/GS were performed as previously described [17, 18].

*NPC1* variants were classified according to the published American College of Medical Genetics and Genomics (ACMG)/Association for Molecular Pathology (AMP) guidelines as P, LP, and variant of unknown significance (VUS) [19, 20].

### Demographic and clinical variables

The regional origin of the patients was categorized into main geographical regions, namely, Europe, Asia, Africa, Middle East, Latin America, and North America. This corresponded to the origin of the requesting clinic/institution.

P/LP variants were grouped into different classes, based on the impact of the mutations at protein function, namely, (1) LoF, (2) missense/conservative, and (3) unknown coding effect. The LoF group included frameshift, canonical splice site, nonsense, and disruptive large deletion/duplication variants. Missense and in-frame deletion/duplication variants were grouped into the missense/conservative category, while synonymous and other intronic variants were grouped into the unknown coding effect group.

### Phenotype analysis

Patients’ phenotype was evaluated using HPO terms, age at diagnosis, and biomarker levels. The HPO terms were classified into main groups: neurological (11 HPO terms), visceral (8 HPO terms), eye related (3 HPO terms), and others (8 HPO terms) (Supplementary Table 1). It should be noted that abnormality of eye movement, namely supranuclear palsy, which corresponds to a neurological abnormality rather than an ophthalmological feature, was among the eye related HPO terms. The age at diagnosis was used as a proxy for age at onset, since the latter was only available for a small group of patients. The age at diagnosis was considered using the distribution into three main groups: below 5 years, between 5 and 18 years, and older than 18 years.

### Biochemical testing

The quantitation of the biomarker PPCS (C_24_H_50_O_7_N_2_P—legacy name lyso SM-509) [14] in DBS was performed by multiple reaction monitoring mass spectrometry (MRM-MS) in positive ion mode on a triple quadrupole mass spectrometer (Sciex 5500) with an ultra-performance liquid chromatography unit (Waters Acquity). The diagnostic cut-off was calculated to be 655 ng/ml, which corresponds to a PPCS/internal standard peak area ratio of 0.9. An extended methods description can be found in the Supplementary information.

### Statistical analysis

Clinical characteristics of the patients identified in the CENTOGENE Biodatabank were summarized as proportions for categorical variables and medians for continuous outcomes.

Comparisons of patients and variant characteristics per demographic variables were done using proportions, and the significance was tested using chi-square test.

The phenotypes (age at diagnosis, biomarker levels, and HPO terms) were compared with main variables, namely: geographical regions, impact of the variant (LoF, missense, in-frame deletion/duplication, synonymous, intronic), and variant frequency for the most common variants. The difference between the phenotypes and the variables was tested using the Kruskal–Wallis test, and *p* values for pairwise comparisons were estimated after correcting for multiple testing. All shown *p* values have been adjusted for multiple testing.

Finally, multivariable regression analyses were used to test the associations between main HPO phenotype outcomes (presence of neurological, eye, or visceral symptoms) and the most common variants using logistic regressions with the main outcome being presence = 1; absence = 0 (e.g., presence of ocular symptoms = 1; absence = 0). Here the associations were represented as the probability (odds ratios) of having a clinical outcome (HPO terms) for patients with a variant vs. patients without the variant. Linear regressions were used to test for associations between age of diagnosis and biomarker levels with the most common variants. Here the significant association is represented as the increase in the mean unit in patients with a variant vs. patients without that variant. The analyses were done using R package v.4.1.1 (https://www.r-project.org/).

## Results

### Patient characteristics

The demographics of the cohort of 602 NPC1 patients are described in Table 1. A slightly higher number of male patients were diagnosed (*N* = 298, 49.5%), compared to female patients (*N* = 257, 42.7%). For the rest (*N* = 47, 7.8%), gender information was not included in the requisition form. Information regarding age at onset of symptoms was not provided by most referring doctors. Thus, the age at diagnosis was recorded as a proxy of disease subtype and severity. Most patients were younger than 5 years, corresponding to the neonatal/infantile forms (*N* = 266, 44.2%), and the median age at diagnosis was 10.6 years (range 0–65 years).

**Fig. 1:**
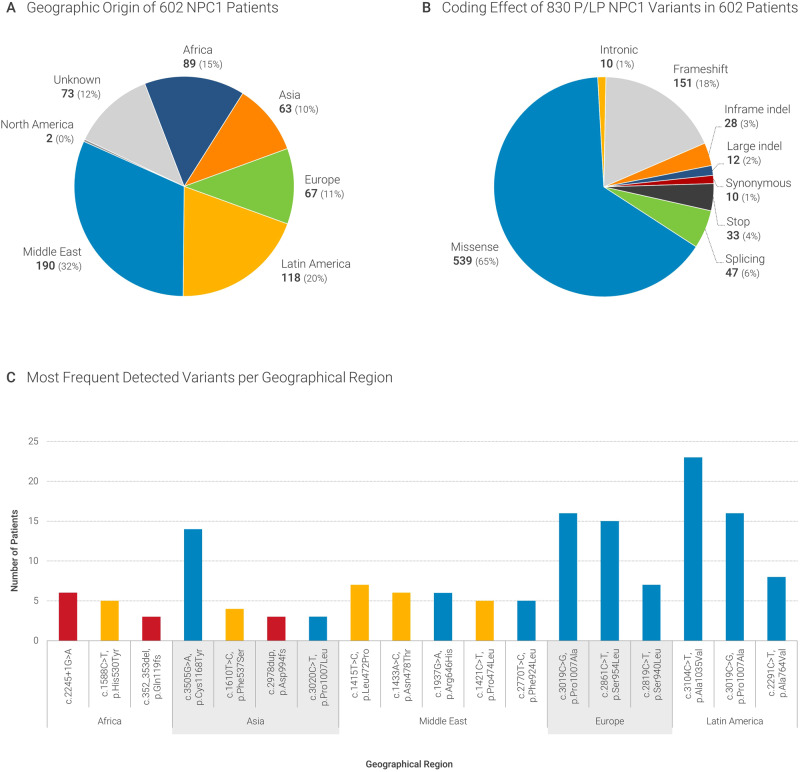
Geographical origin and type of variants. **A** Geographical origin of 602 NPC1 patients (**B**) and coding effect of the 830 P/LP variants detected. **C** Most frequent variants in this cohort and distribution according to geographical origin (red color - LoF variants, orange - variants located in the middle luminal NPC1 domain, blue - other missense variants).

The patients were referred from 47 countries, mostly from the Middle East (*N* = 190, 32%) and Latin America (*N* = 118, 20%), followed by Africa (*N* = 89, 15%) and Europe (*N* = 67, 11%) (Fig. 1A). For most patients, targeted gene sequencing (*N* = 244, 40%) or gene panels (*N* = 214, 35%) were requested (Table 1). The consanguineous status of the parents of patients was provided for 83 patients, with 63 of them being offspring to consanguineous parents. The distribution of consanguinity per country of origin was therefore not assessed.

HPO terms were retrieved from the CENTOGENE Biodatabank in 456 patients. HPO terms had been previously extracted and curated using the clinical information provided in the requisition form and registered in the LIMS by a dedicated team.

The median biomarker level was 1650 ng/ml blood (IQR: 1077-2816). Almost all patients had pathological biomarker levels (cut-off at 655 ng/ml), except for 33 patients who showed normal levels of the biomarker. Among these cases there was no overrepresentation of a particular variant or variant type, geographical origin, or age/year at diagnosis. We noted that approximately half of these cases had borderline upper levels of biomarker (576–655 ng/ml). Repetitions could not be done at a later age or using a new sample.

### Variant characteristics

We identified 830 P/LP variants in 602 patients from our Biodatabank. Of these, 287 were unique and 73 were novel variants (previously unpublished). Most of the novel variants were detected in patients from the Middle East and Latin America (Fig. 1A). Figure 1B shows the coding impact of the P/LP variant occurrences, with 65% of them being missense variants, followed by frameshift (18%) and splicing variants (6%). Most of the variants were detected only in a few cases, with 20% of variants being detected only once. These were seen often in patients from African countries. The most common variants were: c.3019C > G, p.Pro1007Ala (hereafter: p.P1007A) in 48 patients (6%), followed by c.3104C > T, p.Ala1035Val (hereafter: p.A1035V) in 33 patients (4%), c.2861C > T, p.Ser954Leu (hereafter: p.S954L) in 29 patients (3.5%), c.3557G > A, p.Arg1186His (hereafter: p.R1186H) in 19 patients (2.3%), c.3182T > C, p.(Ile1061Thr) (hereafter: p.I1061T) in 18 patients (2.2%), and c.352_353del p.(Gln119fs) (hereafter: p.G119fs) in 17 patients (2%). The newly detected P/LP variants, the clinical data of these patients (HPO terms), as well as biomarker results can be found in Supplementary Table 4.

We looked at the prevalence of the variants per geographical origin of the patients. There were 73 variants (12%) for which the origin was unknown. The most common disease-causing variant of the complete dataset p.P1007A was also the most common variant detected in patients from Europe. The Top2 variant p.A1035V was the most frequent variant detected in patients from Latin America, and the Top3 p.S954L was the second most commonly detected in patients from Europe. Two of the most common variants in patients from Africa were unique to this continent and accounted for 11% of all variants in this continental region (c.2245 + 1G > A; 6% and p.H530Y; 5%), while 32% of the remaining variants were unique. There was an evident clustering of *NPC1* variants per geographical region of origin of the patients. The most common variants per region are present in Fig. 1C.

### Phenotype—HPO terms

We identified 27 HPO terms from the 31 terms known as associated with NPC1 (https://hpo.jax.org/app/browse/gene/4864) (Supplementary Fig. 1). The most common clinical sign reported was abnormal abdomen morphology (*N* = 308, 67.5%, mainly included the terms “hepatomegaly”, “splenomegaly”, “hepatosplenomegaly” and “ascites”). The second most reported clinical term was neurodevelopmental abnormality (*N* = 231, 50.7%, mainly included “developmental regression”, “intellectual disability” and “neurodevelopmental delay”), followed by abnormality of movement (*N* = 143, 31.4%, mainly included “abnormality of movement”, “dystonia”) and abnormality of eye movement (*N* = 119, 26.1%, mainly included “vertical supranuclear gaze palsy”, “ophthalmoplegia”, “upgaze palsy”). We further classified the terms into neurological, eye, visceral abnormalities, and “other” for further analysis (“Materials and methods” and Supplementary Table 1). When interrogated according to the different age categories, 58% of the patients with visceral abnormalities were younger than 5 years; this proportion decreased with age, with this HPO term being reported in only 5% of the patients older than 18 years (Supplementary Fig. 2). When looking at the HPO terms individually, this was also evident as 62% of the patients, with abnormal liver morphology were younger than 5 years vs. 7% in the group older than 18 years. On the other hand, abnormality of movement was found in 59% of older patients (>18 years) vs. 21% in younger patients (<5 years). Abnormality of the coordination was reported in 57% of the patients older than 18 years vs. 10% in the younger patients (<5 years). Abnormality of eye movement was reported in 47% of the patients older than 18 years, while this HPO term was reported in only 15% of the patients younger than 5 years (Supplementary Fig. 3). Clinical summary of selected patients with novel P/LP *NPC1* variants can be found in the Supplementary Information.

### Exploring biomarker levels, age at diagnosis, and genetic variants

To gain insight into the use of “age at diagnosis” as a proxy of “age at onset of symptoms”, we compared the former with the type of variant detected (LoF, missense/conservative, unknown). As expected, patients with LoF variants had a significantly earlier age at diagnosis compared to patients with other types of variants (Supplementary Fig. 4).

**Fig. 2:**
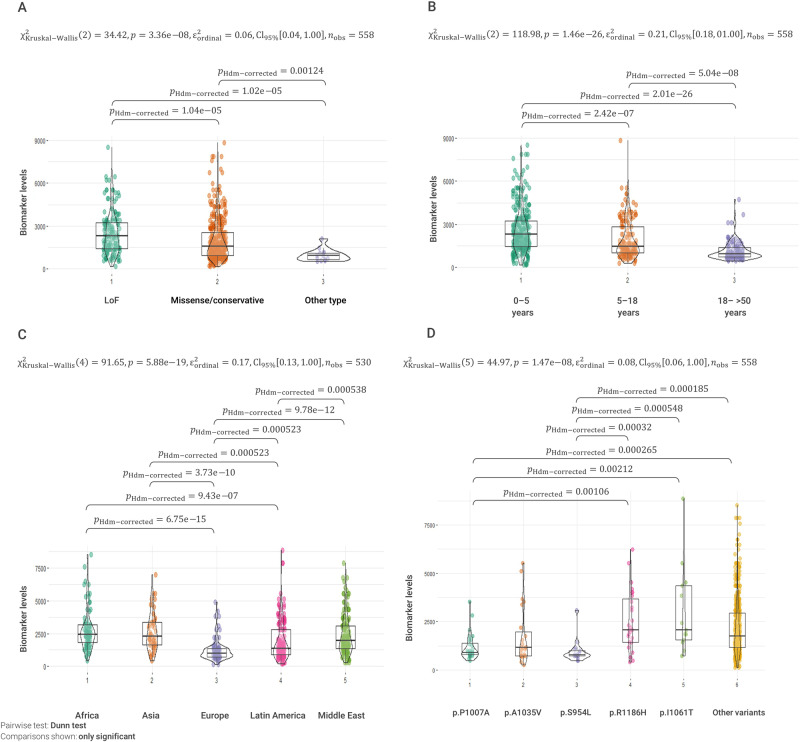
Biomarker PPCS levels (ng/ml) and age at diagnosis, type of NPC1 variants, and region of origin. **A** Patients with LoF variants have significantly higher biomarker results compared to patients with missense or conservative changes, and with variants with unknown effect. **B** Patients with earlier age at diagnosis present significantly higher biomarker values. **C** Patients from Africa, Asia, and the Middle East have significantly higher biomarker values compared to patients from Europe and Latin America. **D** Biomarker levels in patients with the most frequent variants detected in this cohort. Patients with variants p.P1007A, p.A1035V, and p.S954L had significantly lower biomarker levels compared to patients with variants p.R1186H and p.I1061T.

Comparing the type of variant (coding effect) and biomarker levels also lead to significant differences. The patients with LoF variants had higher biomarker levels compared to patients with missense variants (*p* = 1.04E−05) and to patients with unknown effect variants (*p* = 1.02E−05, Fig. 2A).

We found that patients with an age at diagnosis younger than 5 years old had higher biomarker values than older patients (with age at diagnosis between 5–18 years and >18 years, *p* = 2.42E−7 and *p* = 2.01E−26, respectively). Therefore, biomarker PPCS levels were inversely proportional to age at diagnosis (Fig. 2B).

Since we observed geographical clustering of the *NPC1* variants, we explored the patients’ characteristics according to their region of origin. Firstly, we looked at the relationship between age at diagnosis and region of origin. Remarkably, patients from Africa, Asia, and the Middle East presented a significant earlier age at diagnosis compared to patients from Europe and Latin America. The median age at diagnosis was 3 years (IQR: 1–5 years) for Africans, 3 years (IQR: 0.5–6) for Asians, 4 years (IQR: 1–10) for patients from the Middle East, 9 years (IQR: 4–24 years) for Latin Americans, and 14 years (IQR: 6–31 years) for Europeans (Supplementary Fig. 5).

We also compared biomarker levels among the patients from different geographical regions. Higher median levels of biomarkers were found in the patients referred from Africa and Asia, followed by patients from the Middle East, Latin America, and Europe. The median distribution of biomarker levels from European patients was significantly lower than any of the other regions (Fig. 2C).

### HPO terms and most common variant associations

To better understand the relationship between *NPC1* variants and the phenotype of the patients, we analyzed the most common variants using logistic regression models with the HPO phenotype as the outcome (present = 1; absent = 0). We focused on three main group of symptoms, namely having eye abnormalities (yes: 191; no: 417), neurological abnormalities (yes: 491 no: 117), and visceral abnormalities (yes: 405; no: 203).

The variant p.I1061T (Top5) was strongly associated with eye-related abnormalities (Odds ratio = 23.09, *p* = 0.0029, Supplementary Table 3). The HPO terms included in this category were comprised mainly of “abnormality of eye movement”, which was included in this category to be able to differentiate from the broad category of neurological abnormalities (comprised mainly of “neurodevelopmental abnormality” and “abnormality of movement”, Supplementary Table 1). This variant was not significantly associated with either visceral or other neurological symptoms. Other significant associations were found for p.A1035V (Top2) and p.S954L (Top3) with abnormal eye movements (eye-related symptoms). The last variant was also associated with a decreased odds for having visceral symptoms (Odds ratio: 0.16; *p* value = 0.0017, Supplementary Table 3). This means that patients with the p.S954L variant are less likely to present visceral abnormalities but have a higher probability to present abnormal eye movements characteristics of the juvenile form of NPC1 (Supplementary Table 3).

Furthermore, neurological and eye-related abnormalities were associated with later age at diagnosis (*p* = 5.03E−8 and *p* = 1.19E−07, respectively), whereas visceral abnormalities were associated with an earlier age at diagnosis (*p* value < 2.00E−16) (Supplementary Table 2). There was also a trend for LoF variants being more frequent in patients with a visceral phenotype (abnormal abdomen and liver morphology), whereas variants from the “unknown effect” group (synonymous and non-coding) were more frequent in patients with movement abnormalities (Supplementary Fig. 6).

**Fig. 3:**
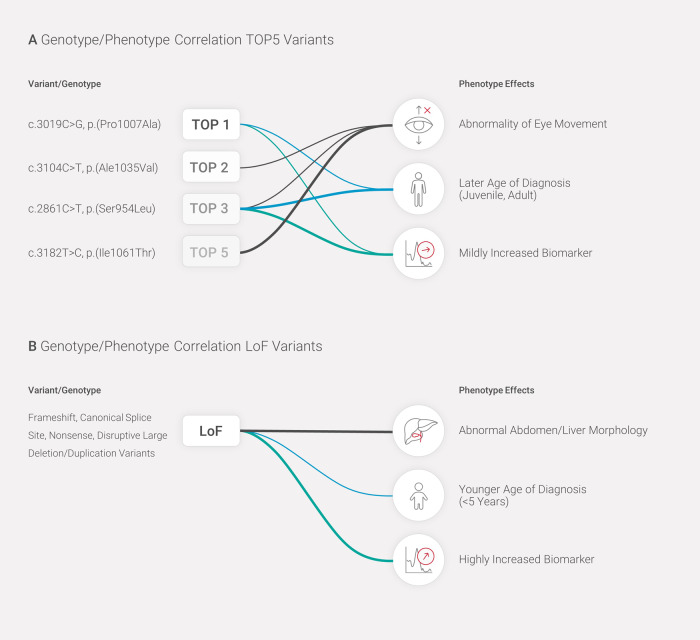
Genotype to phenotype associations with the most frequent variants and LoF variants in this cohort. **A** Significant genotype to phenotype associations for the most frequent variants. **B** Significant genotype to phenotype associations with LoF variants detected. HPO terms, age at diagnosis, and biomarker levels are represented by black, blue, and turquoise lines, respectively. The thickness of the lines represents the strength of the association. All associations are statistically significant after correction for multiple testing.

Finally, we investigated if any of the five most frequent variants in this cohort, namely p.P1007A (Top1), p.A1035V (Top2), p.S954L (Top3), p.R1186H (Top4), and p.I1061T (Top5) were associated with age at diagnosis and biomarker values. We found that patients with the variant p.P1007A (Top1) had a significant later age of diagnosis of 13 years when compared to patients without the variant. Patients that have the p.S954L variant (Top3) had an age of diagnosis 22 years later than patients without the variant (Table 2A). There were no other significant associations for the other “common” variants. The results from the biomarker level analyses were consistent with the former (Table 2B and Fig. 2D). Patients with the p.P1007A (Top1) and p.S954L (Top3) variants showed significantly lower biomarker levels compared to patients without these variants. Specifically, p.P1007A and p.S954L were associated with a reduction of 993 and 1189 ng/ml in the level of the biomarker, respectively, when compared to patients not having these variants. Collectively, these results suggest that both variants usually cause a juvenile/adult form of NPC1 disease. The variant p.R1186H had an opposite effect, with a significant increase of 980.5 ng/ml in the biomarker level in patients with this variant (Table 2B and Fig. 2D). Other associations were not significant. Figure 3 summarizes the most relevant genotype–phenotype associations detected.

**Table 2:** Analyses of most common *NPC1* variants, age at diagnosis and biomarker levels.

A ‘Common’ *NPC1* variants and age at diagnosis			
Variant	Effect size	Standard error	*p* value
**p.P1007A**	**12.7878**	**2.6842**	**2.44e−06**
p.A1035V	−1.1163	3.0885	0.718
**p.S954L**	**21.6224**	**3.3968**	**4.16e−10**
p.R1186H	−1.8628	4.2268	0.66
p.I1061T	5.9438	4.4223	0.18
B ‘Common’ *NPC1* variants and biomarker levels			
	Estimate	Standard error	*p* value
**p.P1007A**	**−992.99**	**287.34**	**0.0006**
p.A1035V	−415.12	318.44	0.193
**p.S954L**	**−1188.6**	**376.54**	**0.002**
**p.R1186H**	**980.5**	**418.24**	**0.01**
p.I1061T	60.66	456.07	0.894

Significant associations are highlighted in bold.

(A) Age at diagnosis and specific *NPC1* variants (from the Top5 most frequent variants). Association analysis between age at diagnosis and presence of a variant; the effect size represents the increase in the mean age in patients having the variant (most of them are homozygotes) versus patients not having the variant. Significantly later age at diagnosis was observed in patients with the p.P1007A and p.S954L variants. (B) Biomarker levels and specific NPC1 variants. Association analysis between level of biomarker and presence of a variant; the effect size represents the increase in the mean biomarker level in patients with the variant (most of them are homozygotes) versus patients without the variant. Significantly lower biomarker levels were observed in patients with the p.P1007A and p.S954L variants, while higher biomarker levels were observed in patients with the p.R1186H variant.

## Discussion

We describe a cohort of 602 NPC1 patients from 47 countries as identified in the CENTOGENE Biodatabank. The patients were genetically diagnosed (biallelic P/LP *NPC1* variants) in our laboratory over the past 15 years. To date, this is so far the largest dataset described for NPC1 disease. Other large cohorts include a study from the United Kingdom that describes 114 patients with *NPC1* causative variants [4]. A recent study from the International Niemann-Pick Disease Registry (INPDR) described 97 patients having *NPC1* variants [21]. Additionally, an Italian cohort of 105 NPC1 patients was recently published [22].

Within our study, the genetic diagnosis of the patients was done using a combination of genetic and biomarker testing whenever blood samples were provided. We confirmed the high allelic heterogeneity of *NPC1* with 287 unique P/LP variants identified. Importantly, 73 of these are novel, unpublished causative variants. This high number of novel variants is likely due to the inclusion of patients from populations that are usually underrepresented in the scientific literature and public genetic databases (e.g., Africa, the Middle East, Latin America).

Although most of the variants identified in the database were rare, with ~21% (*n* = 157) being unique to a single patient, we identified six recurrent variants. The most frequent variant in our cohort was p.P1007A, which was detected in 48 patients (5.8%). This is a known pathogenic variant which leads to the “variant” biochemical phenotype [23–25], mainly with adult disease onset [13, 25, 26]. It has been reported as the second most frequent causative *NPC1* variant [21, 22]. Accordingly, in our cohort, this variant was associated with a later age at diagnosis and lower biomarker levels (Fig. 3). The second most frequent variant in our cohort was p.A1035V (Top2, 4%). The variant has been previously reported in Portuguese and Brazilian patients, and it is characterized as causing a classical phenotype [25, 27]; in our cohort this was the most frequent variant detected in patients from Latin America. The p.S954L variant (Top3, 3.5%) has been described in patients with adult onset of neurological symptoms (ataxia, supranuclear palsy, psychiatric manifestations) [4, 13, 28, 29]. The p.R1186H variant (Top4) has been reported as the most frequent variant in patients from Greece, and it is associated with the classical filipin staining form [26]. It has also been reported in patients from the Czech Republic as causing a severe phenotype [30] and a severe reduction of the NPC1 protein [31], with pronounced abnormalities of cellular cholesterol processing [26, 32]. Our biomarker data is consistent with these previous observations, as patients with this variant in our cohort presented significantly higher biomarker levels (Table 2B and Fig. 2D). The p.I1061T variant was the Top5 in our cohort and Top2 among tested European patients; this variant has been reported as the most frequent causative variant, and it is thought to represent 15–20% of all human *NPC1* disease alleles [24, 33].

Biomarker PPCS levels were significantly associated with age at diagnosis. Younger diagnosed patients (<5 years old) had higher biomarker levels. These patients with a young age at diagnosis and higher biomarker levels also more frequently presented LoF variants and a visceral (abdomen/liver) phenotype. This suggests that the biomarker PPCS could serve as an indicator of disease severity. This observation is in line with a previous report of 36 NPC1 patients with 73 measurements analyzed and showed that the biomarker PPCS significantly correlated with the annual severity increment score [15]. Recently, a study using primary fibroblasts from a cohort of 41 NPC1 patients validated the lysosomal quantitative probe LysoTracker as a predictor for age of onset and disease severity [34]. Additionally, the study also correlated expression of specific genes with clinical age of onset, neurological disease severity, and LysoTracker levels—highlighting the importance of such datasets as an important resource to guide future studies on NPC disease [34].

The compilation of variants in this cohort together with the demographic, clinical, and biomarker characteristics allowed us to identify interesting patterns. Traditionally, genotype to phenotype analysis is patient-centric (patients are considered clinical entities). In this study, we dissected the patients’ phenotypes using the HPO terms related to each patient’s clinical presentation. Although the age at diagnosis of most patients in the cohort is below 5 years of age (44%), 23% of the patients were between 5–18 years old at the time of the diagnosis and 15% of the cohort was diagnosed during adulthood—demonstrating that the known clinical and age-related variability of the NPC1 phenotype is represented in our cohort.

The variant p.S954L was significantly associated with eye-related phenotypes (mainly abnormality of eye movement), to a later age at diagnosis, and to lower biomarker levels (Fig. 3). This confirms previous reports of this variant in patients with late onset neurological presentations, including supranuclear palsy [13, 28]. Additionally, it is in line with the previous report of moderate levels of NPC1 protein, which co-localized with a late endosomal/lysosomal marker and suggested that the mutant protein have residual functionality in cells from four patients with this variant and late disease onset [31]. The variant p.P1007A known as the second most frequent causative variant (Top1 in our cohort) was significantly associated with a later age at diagnosis and a milder biomarker level. This is consistent with previous reports that classify this variant as causing a “variant” biochemical phenotype [23, 25, 35] and mainly adult-onset disease [13, 25, 26]. The most reported *NPC1* variant p.I1061T (Top5, 2.2%) was associated with an eye phenotype (abnormality of eye movement) and detected mainly in patients from Latin America. This variant has been reported to account for 20–25% of alleles in patients diagnosed in France [24] and the United Kingdom [4], but it seems to be much less frequent in countries in southern Europe, with a frequency of 5–10% in Italy and Spain [36]—showing a gradient of increasing frequency from southeast to northwest Europe [22, 32]. This variant has been associated with a juvenile-onset neurological disease with classic biochemical phenotype [24]. Patients are described as having a homogeneous phenotype, with insidious onset of the neurological disease with learning problems in school, followed by intellectual disability later in life. The course of the disease is slow with cerebellar involvement, dystonia, and vertical supranuclear ophthalmoplegia [24].

We noticed clustering of certain *NPC1* variants per geographic area (Fig. 1C) and investigated whether these patients exhibited different clinical or biomarker features. Variants detected in patients from Africa, Asia, and the Middle East were associated with an earlier age at diagnosis (3–4 years vs. 9–14 years) compared to variants in tested patients from Latin America and Europe and to higher biomarker values. Accordingly, these variants were mainly LoF and missense variants located in the middle luminal domain (MLD, Fig. 1C). Previous studies have shown the functional relevance of the MLD of the NPC1 protein (aa 385–607) which binds NPC2 in vitro and in vivo [37, 38]. It is generally accepted that premature termination codon variants, variants involving the Sterol-Sensing Domain (SSD) (aa 620–785), and p.A1054T in the cysteine-rich luminal loop of NPC1 are associated with early-infantile NPC1 [35].

The three most common *NPC1* variants in our cohort are associated with lower biomarker levels and later age at diagnosis: p.P1007A, p.A1035V, and p.S954L. These are also the most frequently identified variants in tested patients from Europe and Latin America. Therefore, the differences among clinical presentations of patients from these regions might be related to the specific variants detected in patients from each area.

Limitations of the study: the inclusion of patients in our Biodatabank relies on referrals from many centers and doctors (referral bias), therefore the geographical distribution of patients and variants in our study might be not representative. Unfortunately, we do not have sufficient data related to the age of onset of symptoms and the natural history of the disease, nor do we have clinical information to establish a scale of severity of the disease. Furthermore, we must flag that the clinical assessment for NPC1 patients might greatly differ in different countries due to awareness, availability of diagnostic resources, and training of health care professionals impacting the setting in which children and adults with suspected neurometabolic disorders gained access to molecular testing offers.

In conclusion, we describe the largest and most heterogenous cohort of NPC1 patients published to date, including 73 novel P/LP variants, expanding *NPC1* allelic heterogeneity. In addition to the diagnostic value of the biomarker PPCS, which is especially valuable in patients with missense variants, the biomarker might be useful to indicate disease severity/progression. Additionally, we confirmed previous genotype–phenotype associations and established novel genotype-HPO term-biomarker relationships for the most frequent *NPC1* variants.

### Supplementary information

Supp material

## Data Availability

The dataset that was generated and/or analyzed as part of this study is available from the corresponding author. All novel pathogenic and likely pathogenic variants have been deposited in ClinVar (https://www.ncbi.nlm.nih.gov/clinvar/?term=centogene+npc1).
