# Supplementary material for: At a glance: the largest Niemann-Pick type C1 cohort with 602 patients diagnosed over 15 years
Source: Eur J Hum Genet. 2023 Jul 11;31(10):1108–16. doi: 10.1038/s41431-023-01408-7 (PMC10545733; doi:10.1038/s41431-023-01408-7)

**Supplementary Information**

**Extended Methods**

Biochemical testing

The structure of the biomarker PPCS-509 (N-palmitoyl-O-phosphocholineserine [PPCS], C24H50O7N2P)^16^ was verified by comparative Liquid chromatography-high resolution mass spectrometry (LC-HRMS/MS) analysis of endogenous and synthesized compound. Both compounds show the same mass to charge, retention time, collision cross section, and fragment ions respectively. The quantitation of the biomarker PPCS (legacy name lyso SM-509) (m/z = 509.3354 for [M+H]+) in dried blood spots was performed by multiple-reaction-monitoring mass spectrometry (MRM-MS) in positive ion mode on a triple quadrupole mass spectrometer (Sciex 5500) with an ultra-performance liquid chromatography unit (Waters Acquity). Before the PPCS structure was elucidated, all tests were clinically validated according to ISO 15189 guidelines using a semiquantitative method based on the peak area ratio of analyte to internal standard. After the PPCS structure determination and confirmation, the synthesized compound was used to prepare a calibration line to retrospectively calculate the PPCS concentration levels from the analyte/internal standard peak area ratio in all historically measured DBS samples.

A series of 13 PPCS dilutions between 50 and 9600 ng/mL were measured in 14 replicates across four different days by two different operators, showing a high reproducibility at each concentration point with a respective CV< 20%. The calibration curve displays a linear range up to 1200 ng/mL. By using a saturation fitting equation, it was possible to extend the analytical measurement range (AMR) to 9600 ng/mL. The deviations between the fitting curve and the measured values are below 10% for the two lowest concentration points and below 4% for all higher concentration points even for those that are beyond the linear range. The diagnostic cut-off was calculated to be 655 ng/mL, which corresponds to a PPCS/internal standard peak area ratio of 0.9.


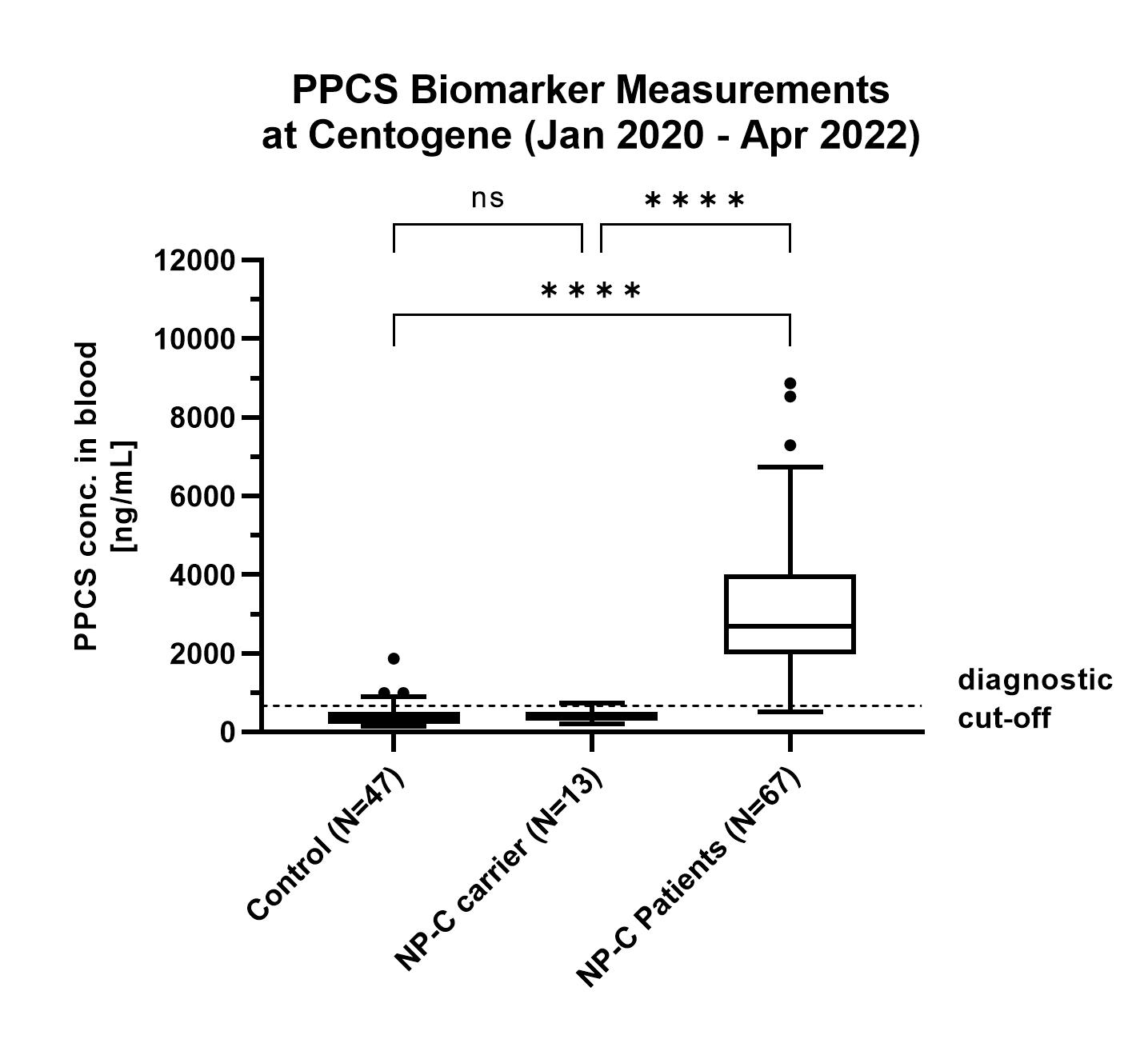


**Selected case reports**

**Case report 1**

The index is a young child born to consanguineous parents, with two healthy siblings. The index was born by C-section after a normal pregnancy. She had low birth weight. She presented with jaundice form the age of 2-month-old. The patient presented feeding difficulties with food intolerance, leading to failure to thrive. She was hospitalized on several occasions because of aspiration pneumonia. The patient had global developmental delay that was evident since the 3^rd^ month of life. Physical examination at 2-years-old showed mild dysmorphic features, mild hepatosplenomegaly, hypertonia, hyperreflexia, and clonus. Abdominal ultrasound confirmed hepatosplenomegaly and detected mesenteric lymphadenitis. Liver enzymes and cholesterol were elevated. Genetic testing (panel) detected a homozygous pathogenic variant in *NPC1*, c.1180_1181dup, p.Phe395fs, with pathologically elevated biomarker, establishing the genic diagnosis of Nieman Pick type C.

**Case report 2**

The index is a pediatric male patient born to consanguineous parents, with two siblings: a younger sister deceased at the age of 2 years, and an asymptomatic sibling. The index presented pallor, marked hepatosplenomegaly, with distended abdomen. He presented ataxia as well. Genetic testing (panel) identified a homozygous pathogenic variant in *NPC1* NM_000271.4:c.2758G>A p.(Val920Met) and pathologically increased biomarker levels, with the genetic diagnosis of Nieman Pick type C.

The same variant was identified in an unrelated patient (1-year-old) with protuberant abdomen, hepatosplenomegaly, mild global developmental delay, and pathologically increased biomarker.

**Case report 3**

The index is a 61 years-old female patient born to nonconsanguineous parents. The patient presented with seizures, ataxia, movement abnormalities, cerebellar dysfunction and ophthalmoplegia. She had also intellectual disability. Genetic testing (exome sequencing) revealed a homozygous pathogenic *NPC1* variant NM_000271.4:c.574T>C, p.(Tyr192His), and pathologically increased biomarker. The same variant had been identified in another patient in compound heterozygosity with the pathogenic variant NM_000271.4:c.1433A>C, p.(Asn478Thr). The patient was 20 years old at the time of the diagnosis and presented generalized dystonia and vertical gaze palsy.

**Supplementary tables and Figures**

**Supplementary Table 1.** The HPO terms were classified into main groups: ocular related (3 HPO terms – group 1), neurological (11 HPO terms - group 2), visceral (8 HPO terms – group 3), and others (8 HPO terms -group 4). Note that ‘Abnormality of eye movement’ was assigned to group 1 to be able to differentiate this specific clinical sign from the broad neurological group which comprised mainly neurodevelopmental delay and abnormality of movement.

| HPO term | Analysis group |
| --- | --- |
| Abnormality of eye movement | 1 |
| Optic atrophy | 1 |
| Cherry red spot of the macula | 1 |
| Behavioral abnormality | 2 |
| Seizure | 2 |
| Failure to thrive | 2 |
| Growth delay | 2 |
| Neurological speech impairment | 2 |
| Abnormality of coordination | 2 |
| Abnormal nervous system morphology | 2 |
| Neurodevelopmental abnormality | 2 |
| Abnormal reflex | 2 |
| Abnormality of movement | 2 |
| Cognitive impairment | 2 |
| Decreased liver function | 3 |
| Abnormal abdomen morphology | 3 |
| Hyperbilirubinemia | 3 |
| Elevated hepatic transaminase | 3 |
| Foam cells | 3 |
| Abnormality of the biliary system | 3 |
| Abdominal symptom | 3 |
| Abnormal liver morphology | 3 |
| Abnormality of blood and blood forming tissues | 4 |
| Respiratory tract infection | 4 |
| Abnormality of the skeletal system | 4 |
| Dysphagia | 4 |
| Abnormal lung morphology | 4 |
| Respiratory insufficiency | 4 |
| Dyspnea | 4 |
| Abnormal muscle tone | 4 |

**Supplementary Table 2.** Association between HPO terms and age at diagnosis. Patients with neurological and eye related abnormalities had a later age at diagnosis, while patients with a visceral phenotype were diagnosed at an earlier age.

| HPO term category | Effect size and standard error  Age at diagnosis | P-value |
| --- | --- | --- |
| Neurological abnormalities | 0.12 (0.02) | 1.19e-07 |
| Eye abnormalities^1^ | 0.04 (0.007) | 5.03 e-8 |
| Visceral abnormalities | -0.14 (0.013) | <2e-16 |

**^1^** Mainly abnormal eye movement (of neurological cause)

**Supplementary Table 3.** Genotype to phenotype associations with the most frequent variants in this cohort and the main HPO term categories. The odds ratio presents the probability (odds) of having a phenotypic outcome (HPO terms) in patients with the variant (most of them are homozygous) versus patients without the variant. Significant associations are **in bold**. *The high odds ratio is the result of the low number of individuals per group and therefore the estimate is not reliable.

| **Eye related abnormalities** | | | | **Neurological abnormalities** | | | **Visceral abnormalities** | | |
| --- | --- | --- | --- | --- | --- | --- | --- | --- | --- |
| Variant | Effect (sterr) | Odds.Ratio | P-value | Effect (sterr) | Odds.Ratio | P-value | Effect (sterr) | Odds Ratio | P-value |
| p.P1007A | 0.68 (0.39) | 1.97 | 0.08 | 1.91 (1.02) | 6.72 | 0.06 | -0.88 (0.39) | 0.42 | **0.02** |
| **p.A1035V** | 0.82 (0.42) | 2.27 | **0.05** | 0.99 (0.75) | 2.69 | 0.19 | 0.01 (0.44) | 1.01 | 0.99 |
| **p.S954L** | 1.07 (0.51) | 2.91 | **0.04** | 1.29 (1.04) | 3.64 | 0.21 | -1.84 (0.58) | 0.16 | **0.00** |
| p.R1186H | -0.43 (0.66) | 0.65 | 0.52 | 1.06 (1.05) | 2.89 | 0.31 | 0.13 (0.61) | 1.13 | 0.84 |
| **p.I1061T** | 3.14 (1.05) | 23.09 | **0.0029** | 15.15 (723.49)^*^ | 3799195.91* | 0.98 | -0.52 (0.61) | 0.60 | 0.40 |

**Supplementary Table 4**. List of 73 novel P/LP variants in *NPC1(*NM_000271.4) (unpublished, submitted to ClinVar). Genotype Other/complex refers to cases with 3 alleles identified. Biomarker was pathologically increased in all cases, except for the ones marked ^1^ for whom there was not data. Variants in bold were identified in different (unrelated) patients.

| **cDNA** | **Genotypes** | **Protein** | **Variant class** | **HPO TERMS** |
| --- | --- | --- | --- | --- |
| c.1084_1093del | H | p.Thr362fs | P | Hepatosplenomegaly  Premature birth |
| c.1110_1113del | CH | p.Val371fs | P | Hepatosplenomegaly |
| c.1180_1181dup | H | p.Phe395fs | P | Abnormal stool composition  Aspiration  Brisk reflexes  Caesarian section  Clonus  Decreased body weight  Dysphagia  Elevated hepatic transaminase  Enlarged mesenteric lymph node  Epicanthus  Failure to thrive  Food intolerance  Global developmental delay  Hepatomegaly  Hepatosplenomegaly  Hyperreflexia  Hypertonia  Inverted nipples  Jaundice  Microcephaly  Neuromuscular dysphagia  Pneumonia  Scissor gait  Short stature  Small for gestational age  Spasticity  Tremor |
| c.1238dup | CH | p.Leu414fs | P | Ataxia |
| **c.1286T>G** | H | p.Val429Gly | LP | Ataxia  Delayed speech and language development  Developmental regression  Elevated hepatic transaminase  Failure to thrive  Global brain atrophy  Hepatosplenomegaly  Intellectual disability  Motor delay  Seizure |
| **c.1286T>G** | H | p.Val429Gly | LP | Ataxia  Cerebellar atrophy  Delayed speech and language development  Developmental regression  Elevated hepatic transaminase  Failure to thrive  Hepatosplenomegaly  Intellectual disability  Motor delay  Seizure |
| **c.1286T>G** | H | p.Val429Gly | LP | Abnormal circulating creatinine concentration  Abnormality of the skeletal system  Coarse facial features  Global developmental delay Hepatosplenomegaly  Intellectual disability |
| **c.1286T>G** | H | p.Val429Gly | LP | Splenomegaly |
| **c.1286T>G** | H | p.Val429Gly | LP | Ophthalmoplegia  Splenomegaly |
| c.1339C>A | H | p.Gln447Lys | LP | Congenital onset  Hyperargininemia  Hyperlysinemia  Hypermethioninemia  Hyperthreoninemia  Hypertyrosinemia  Muscular hypotonia of the trunk  Neonatal cholestatic liver disease |
| c.1451del | H | p.Val484fs | P | Cholestasis  Hepatomegaly  Proximal muscle weakness  Splenomegaly |
| c.1510_1512del | H | p.Phe504del | LP | Abnormality of movement  Intellectual disability  Seizure  Splenomegaly |
| c.1612_1619dup | Other/complex | p.Phe542fs | P | Cerebellar atrophy  Delayed speech and language development  Failure to thrive  Global developmental delay  Intellectual disability  Muscle weakness |
| c.1615G>A | CH | p.Gly539Arg | LP | Cholestasis  Intracranial hemorrhage |
| c.1654+1dup | H | p.? | P | Clinical suspicion of NPC1 |
| c.1747_1755delinsAT | CH | p.Trp583fs | P | Ataxia  Motor delay  Splenomegaly  Vertical supranuclear gaze palsy |
|  |  |  |  |  |
| c.1757+1G>T | H | p.? | P | Clinical suspicion of NPC1 |
| **c.1757+2_1757+3del** | CH | p.? | P | Ataxia  Brain atrophy  Cerebral palsy  Delayed speech and language development  Developmental regression  Elevated hepatic transaminase  Failure to thrive  Hepatosplenomegaly  Intellectual disability  Motor delay  Seizure |
| **c.1757+2_1757+3del** | CH | p.? | P | Abnormality of movement  Muscle weakness  Ophthalmoplegia  Splenomegaly |
| **c.1757+2_1757+3del** | CH | p.? | P | Abnormal cerebellum morphology  Abnormality of movement  Ataxia  Hepatosplenomegaly  Intellectual disability |
| c.1757+2T>G | CH | p.? | P | Ataxia  Brain atrophy  Developmental regression  Dystonia  Seizure  Spasticity  Splenomegaly |
| c.1780_1782del | CH | p.Tyr594del | LP | Abnormality of movement  Cataplexy  Intellectual disability  Seizure |
| c.2027G>T | H | p.Ser676Ile | LP | Hepatosplenomegaly  Prolonged neonatal jaundice |
| c.2077del | H | p.Leu693fs | P | Abnormality of eye movement  Global developmental delay  Hepatosplenomegaly |
| c.2086del | CH | p.Ala696fs | P | Clinical suspicion of NPC1 |
| c.2130+1G>C | H | p.? | P | Abnormal circulating creatine kinase concentration  Abnormal myelination  Developmental regression  Global developmental delay  Hyperreflexia  Muscular hypotonia  Pseudobulbar paralysis |
| c.2134_2135del | H | p.Asp712fs | P | Generalized hypotonia  Intellectual disability  Muscle weakness  Splenomegaly |
| c.2146C>T | CH | p.Gln716* | P | Abnormality of movement  Ataxia  Behavioral abnormality  Elevated hepatic transaminase  Intellectual disability  Splenomegaly |
| c.2158_2162del | H | p.Leu720fs | P | Failure to thrive  Hepatomegaly  Neonatal hyperbilirubinemia  Ophthalmoplegia  Splenomegaly |
| c.2515-2A>G | H | p.? | P | Abnormality of eye movement  Developmental regression  Splenomegaly |
| c.2604+2T>G | H | p.? | P | Neurodegeneration |
| c.2612del | CH | p.Tyr871fs | P | Failure to thrive  Global developmental delay  Hepatomegaly  Hepatosplenomegaly  Infantile onset  Neonatal hyperbilirubinemia  Prolonged neonatal jaundice  Seizure  Splenomegaly  Supranuclear gaze palsy |
| c.2647del | H | p.Leu883fs | P | Behavioral abnormality  Failure to thrive  Global developmental delay  Hepatomegaly  Prolonged neonatal jaundice  Splenomegaly  Vomiting |
| c.2695T>C | H | p.Tyr899His | LP | Cataplexy  Developmental regression  Muscle weakness  Splenomegaly |
| c.2719del^1^ | H | p.Met907fs | P | Congenital onset  Conjugated hyperbilirubinemia  Hepatosplenomegaly |
| c.2736_2751del | H | p.Met912fs | P | Hepatosplenomegaly  Motor delay  Prolonged neonatal jaundice |
| c.2758G>A | H | p.Val920Met | LP | Protuberant abdomen  Splenomegaly |
| c.2758G>A | H | p.Val920Met | LP | Abdominal distention  Ataxia  Childhood onset  Gait ataxia  Hepatomegaly  Pallor  Splenomegaly |
| c.2770T>C | H | p.Phe924Leu | LP | Cherry red spot of the macula  Jaundice |
| c.2782C>T | CH | p.Gln928* | P | Abnormal larynx morphology  Abnormality of the musculature of the thigh  Arthralgia  Ataxia  Failure to thrive in infancy  Global developmental delay  Hepatosplenomegaly  Intellectual disability  Neurological speech impairment  Poor speech  Skin nodule  Upgaze palsy |
| c.2882_2897delinsG | CH | p.Asn961_Phe966delinsSer | LP | Splenomegaly |
| c.2908dup | H | p.Ser970fs | P | Abnormality of bone marrow cell morphology  Central hypotonia  Failure to thrive  Global developmental delay  Hepatomegaly  Splenomegaly |
| c.3003del | H | p.Met1001fs | P | Delayed speech and language development  Developmental regression  Intellectual disability  Motor delay  Muscular hypotonia  Splenomegaly |
| c.3032_3038delinsAGGTTTACTC | H | p.Cys1011_Lys1013del  insTer* | P | Abnormality of eye movement  Failure to thrive in infancy  Global developmental delay  Hepatomegaly  Prolonged neonatal jaundice  Seizure  Splenomegaly  Supranuclear gaze palsy |
|  |  |  |  |  |
| c.3041+1G>T | H | p.? | P | Abnormality of the gallbladder  Delayed speech and language development  Developmental regression  Difficulty walking  Failure to thrive  Hepatomegaly  Intellectual disability  Motor delay  Mutism  Splenomegaly |
| c.3096_3116delinsCAGGTCG | H | p.Arg1032fs | P | Abnormality of glycosphingolipid metabolism  Dysphagia  Hepatosplenomegaly  Infantile onset  Osteoporosis  Recurrent respiratory infections  Recurrent upper respiratory tract infections |
| c.3154_3156del | H | p.Ile1052del | LP | Abdominal distention  Abnormal posturing  Abnormality of the periventricular white matter  Anemia  CNS demyelination  Caesarian section  Decreased mean corpuscular hemoglobin concentration  Decreased mean corpuscular volume  Decreased serum creatinine  Dystonia  Elevated alpha-fetoprotein  Elevated serum aspartate aminotransferase  Enlarged mesenteric lymph node  Extra-axial cerebrospinal fluid accumulation  Failure to thrive  Generalized-onset seizure  Global developmental delay  Hepatic hemangioma  Hepatomegaly  Hepatosplenomegaly  Hyperintensity of cerebral white matter on MRI  Hyperreflexia  Hypochloremia  Motor delay  Prolonged partial thromboplastin time  Prolonged prothrombin time  Reduced blood urea nitrogen  Reduced hematocrit  Seizure  Splenomegaly  Ventriculomegaly |
| c.3245+1G>T | H | p.? | P | Ataxia  Muscular hypotonia  Splenomegaly |
| c.3249_3250del | CH | p.Phe1084fs | P | Abnormal myelination  Abnormality of movement  Ataxia  Behavioral abnormality  Intellectual disability  Muscle weakness  Ophthalmoplegia (Progressive) |
| c.3249_3250del | CH | p.Phe1084fs | P | Abnormality of movement  Ataxia  Behavioral abnormality  Clumsiness  Dementia  Frequent falls  Hallucinations  Supranuclear gaze palsy |
| c.3249_3250del | CH | p.Phe1084fs | P | Ataxia  Cataplexy  Clumsiness  Cognitive impairment  Dementia  Frequent falls  Hepatomegaly  Splenomegaly  Vertical supranuclear gaze palsy |
| c.3249_3250del | H | p.Phe1084fs | P | Developmental regression  Muscle weakness  Pulmonary infiltrates  Splenomegaly |
| c.3265G>C | CH | p.Glu1089Gln | LP | Clinical suspicion of NPC1 |
| c.3281del | CH | p.Ile1094fs | P | Clinical suspicion of NPC1 |
| c.3282_3284del | H | p.Ile1095del | LP | Developmental regression  Gastroesophageal reflux  Intellectual disability |
| c.3314C>A | H | p.Ser1105Tyr | LP | Global developmental delay  Intellectual disability  Seizure  Splenomegaly  Vertical supranuclear gaze palsy |
| c.3347del ^1^ | H | p.Leu1116fs | P | Abnormality of the coagulation cascade  Ascites  Cholestasis  Cholestatic liver disease  Decreased liver function  Failure to thrive  Hepatosplenomegaly |
| c.338G>A | CH | p.Cys113Tyr | LP | Hepatosplenomegaly |
| c.3412_3413del | CH | p.Met1138fs | P | Abnormality of skeletal morphology  Bone pain  Bowel incontinence  Constipation  Diarrhea  Hepatomegaly  Nausea  Progressive neurologic deterioration  Recurrent respiratory infections  Restricted large joint movement  Scoliosis  Seizure  Spastic tetraparesis  Splenomegaly  Vomiting  Weight loss |
| c.3490T>C | CH | p.Ser1164Pro | LP | Behavioral abnormality  Failure to thrive  Global developmental delay  Hepatosplenomegaly  Intellectual disability  Irritability  Prolonged neonatal jaundice |
| c.3559del | Other/complex | p.Ala1187fs | P | Clinical suspicion of NPC1 |
| c.3614C>T | H | p.Thr1205Ile | LP | Abnormality of movement  Ataxia  Dementia  Dysarthria  Intellectual disability  Joint hypermobility  Muscle weakness  Ophthalmoplegia  Splenomegaly  Vertical supranuclear gaze palsy |
| c.370dup^1^ | CH | p.Thr124fs | P | Abnormality of movement  Abnormality of the nervous system  Hepatosplenomegaly  Intellectual disability  Seizure  Splenomegaly |
| c.3732_3735del | H | p.Pro1245fs | LP | Clinical suspicion of NPC1 |
| c.392dup^1^ | CH | p.Asp131fs | LP | Aggressive behavior  Attention deficit hyperactivity disorder  Bruising susceptibility  Developmental regression  EEG abnormality  Echolalia  Inappropriate laughter  Intellectual disability |
| c.428_429del | H | p.Glu143fs |  | Abdominal distention  Abnormal enzyme/coenzyme activity  Anteverted nares  Caesarian section  Coarse facial features  Conjunctival icterus  Decreased body weight  Depressed nasal bridge  Elevated serum alanine aminotransferase  Elevated serum aspartate aminotransferase  Failure to thrive Generalized hypotonia  Hepatomegaly  Hepatosplenomegaly  Hyporeflexia  Intrahepatic cholestasis  Jaundice  Macrotia  Microcephaly  Patent ductus arteriosus  Patent foramen ovale  Reduced number of intrahepatic bile duct  Short stature  Small for gestational age  Splenomegaly  Triangular face |
| c.428_429del | CH | p.Glu143fs | P | Abnormality of movement  Intellectual disability  Vertical supranuclear gaze palsy |
| c.497C>A | CH | p.Pro166His | LP | Intellectual disability  Specific learning disability  Splenomegaly |
| c.529T>C | H | p.Cys177Arg | LP | Abnormality of eye movement  Hepatomegaly  Intellectual disability  Jaundice  Seizure  Splenomegaly |
| c.532G>A | CH | p.Gly178Arg | LP | Failure to thrive  Global developmental delay  Hepatosplenomegaly  Prolonged neonatal jaundice  Seizure  Vertical supranuclear gaze palsy |
| c.551G>C | H | p.Cys184Ser | LP | Agenesis of corpus callosum  Autism  Brain atrophy  Corpus callosum atrophy  Delayed speech and language development  Developmental regression  Encephalopathy  Generalized-onset seizure  Hepatomegaly  Muscular hypotonia  Psychomotor deterioration  Seizure  Splenomegaly |
| **c.574T>C** | CH | p.Tyr192His | LP | Generalized dystonia  Vertical supranuclear gaze palsy |
| **c.574T>C** | H | p.Tyr192His | LP | Abnormal cerebellum morphology  Abnormality of movement  Ataxia  Intellectual disability  Ophthalmoplegia  Seizure  Vertical supranuclear gaze palsy |
| c.692_693del | H | p.Val231fs | P | Hepatosplenomegaly |
| c.749_755del | CH | p.Lys250fs | P | Clinical suspicion of NPC1 |
| c.754del | CH | p.Gln252fs | P | Failure to thrive  Global developmental delay  Hepatosplenomegaly  Prolonged neonatal jaundice  Seizure  Vertical supranuclear gaze palsy |
| c.754del | H | p.Gln252fs | P | Cholelithiasis  Generalized hypotonia  Hepatosplenomegaly |
| c.762del | H | p.Pro255fs | P | Hepatosplenomegaly  Intellectual disability  Neonatal hyperbilirubinemia |
| c.803_805dup | CH | p.Met268_Tyr269insLeu | LP | Ataxia  Cholestasis  Hepatic failure  Mental deterioration  Neonatal onset |
| c.895del | CH | p.Val299fs | P | Abnormal corpus callosum morphology  Childhood onset  Gait ataxia |
| c.924_927del | CH | p.Asn308fs | P | Abnormality of movement  Ataxia  Cataplexy  Hepatosplenomegaly  Intellectual disability  Ophthalmoplegia  Seizure |
| heterozygous deletion encompassing exon 5 and 6 | CH | p.? | P | Abnormality of movement  Dysphagia  Progressive cerebellar ataxia  Splenomegaly |
| heterozygous large deletion encompassing exon 19^1^ | CH | p.? | P | Hepatosplenomegaly  Jaundice |
| heterozygous large deletion encompassing exon 20 | CH | p.? | P | Clinical suspicion of NPC1 |
| heterozygous large deletion encompassing exons 6 to 9 | CH | p.? | P | Cognitive impairment  Hepatomegaly  Horizontal supranuclear gaze palsy  Intellectual disability  Mental deterioration  Paraparesis  Prolonged neonatal jaundice  Splenic rupture  Splenomegaly |
| homozygous deletion encompassing exon 8 to 9 | H | p.? | P | Flexion contracture  Intellectual disability  Muscle weakness  Splenomegaly |

**Supplementary Figure 1**. Clinical spectrum: most frequently reported HPO terms in 456 patients diagnosed with NPC1. Abnormal abdomen morphology (67.5%), Neurodevelopmental delay (50%), Abnormality of movement/coordination (31%) and Abnormality of eye movement (26%) were the most frequently reported HPO terms.


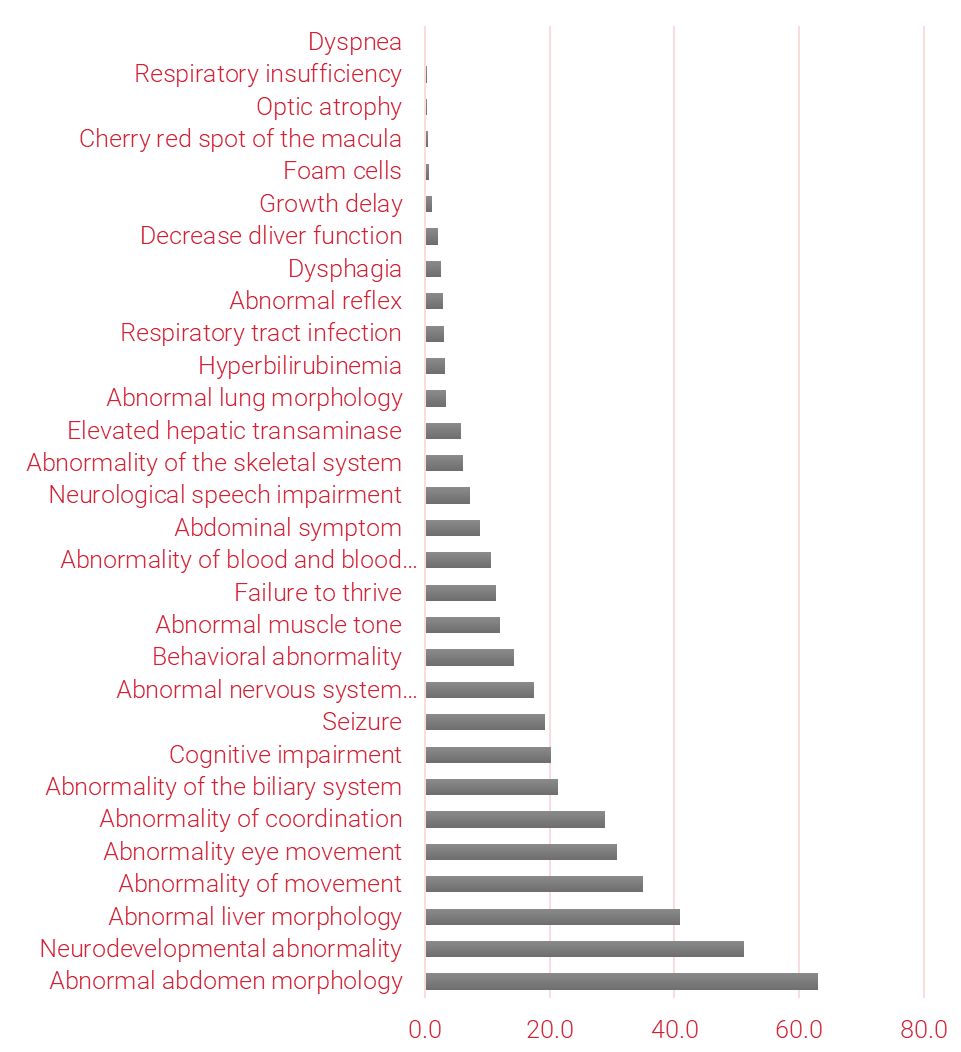


**Supplementary Figure 2.** HPO categories as neurological, visceral and eyes abnormalities per age group (age at diagnosis). Visceral abnormalities were mainly reported in patients younger than 5 years (58%). The percentage of HPO categories from patients unknown age was similar (no bias).

**Supplementary Figure 3.** Main HPO terms per age groups (age at diagnosis). Abnormal liver morphology was reported in 62% of the patients younger of 5 years vs. only 7% in patients older than 18 years. Abnormality of movement (59%) and of coordination (57%) were reported mainly in patients older than 18 years.

**Supplementary** **Figure 4.** Age at diagnosis and type of variant.

**LoF Missense/conservative Unknown**


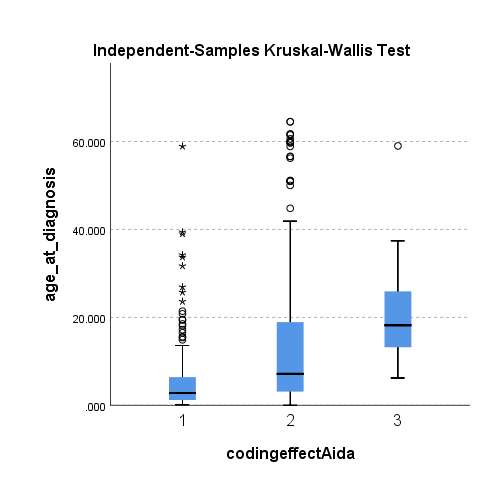


| **Pairwise Comparisons Variants coding effect** | | | | | |
| --- | --- | --- | --- | --- | --- |
| Sample 1-Sample 2 | Test Statistic | Std. Error | Std. Test Statistic | Sig. | Adj. Sig.^a^ |
| LoF -Missense/conservative | -104.157 | 14.750 | -7.061 | .000 | **.000** |
| LoF-Unknown | -224.435 | 45.058 | -4.981 | .000 | **.000** |
| Missense/conservative-Unknown | -120.278 | 44.115 | -2.726 | .006 | .019 |
| Each row tests the null hypothesis that the Sample 1 and Sample 2 distributions are the same.  Asymptotic significances (2-sided tests) are displayed. The significance level is .05. | | | | | |
| a. Significance values have been adjusted by the Bonferroni correction for multiple tests. | | | | | |

**Supplementary Figure 5.** Age at diagnosis per geographical origin of the patients. Patients from Africa, Asia and Middle East presented a significant earlier age at diagnosis compared to patients from Europe and Latin America.


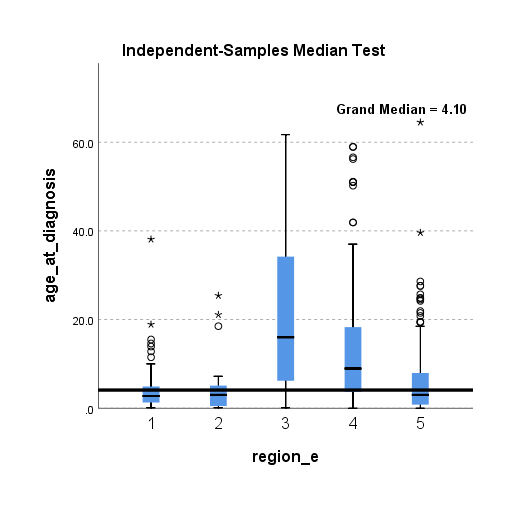


Africa Asia Europe Latin America Middle East

| **Pairwise Comparisons of region of origin**  (1=Africa, 2=Asia, 3=Europe, 4=Latin America, 5=Middle East) | | | |
| --- | --- | --- | --- |
| Sample 1-Sample 2 | Test Statistic | Sig. | Adj. Sig.^a^ |
| 1.00-2.00 | .398 | .528 | 1.000 |
| 1.00-5.00 | .190 | .663 | 1.000 |
| 1.00-4.00 | 30.226 | .000 | .000 |
| 1.00-3.00 | 38.903 | .000 | .000 |
| 2.00-5.00 | 8.889E-5 | .992 | 1.000 |
| 2.00-4.00 | 25.855 | .000 | .000 |
| 2.00-3.00 | 36.686 | .000 | .000 |
| 5.00-4.00 | 24.164 | .000 | .000 |
| 5.00-3.00 | 33.828 | .000 | .000 |
| 4.00-3.00 | 5.010 | .025 | .252 |
| Each row tests the null hypothesis that the Sample 1 and Sample 2 distributions are the same.  Asymptotic significances (2-sided tests) are displayed. The significance level is .05. | | | |
| a. Significance values have been adjusted by the Bonferroni correction for multiple tests. | | | |
|  | | | |

**Supplementary Figure 6.** HPO terms and coding effect of variants. LoF variants are more frequent in the patients with a visceral phenotype (abnormal abdomen and liver morphology), whereas variants from the ‘unknown effect’ group (synonymous and non-coding) are more frequent in patients with movement abnormalities.


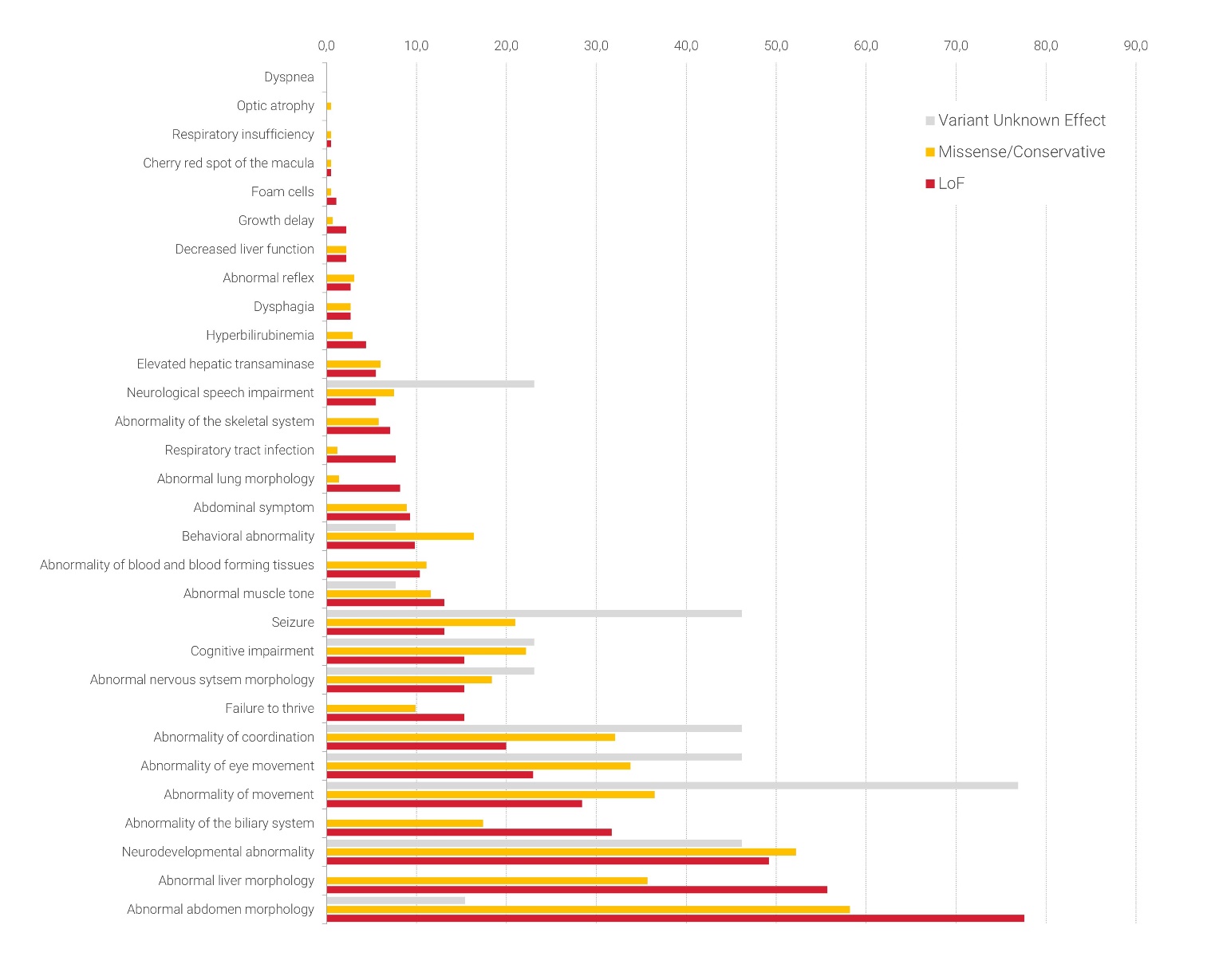

Supplement: Supplementary file 1 — Supp material [file 41431_2023_1408_MOESM1_ESM.docx]
